# Association of ORAI1 Genetic Polymorphism with Serum Calcium and Phosphorus Levels in Non-dialysis Chronic Kidney Disease Patients: A Case-control Study

**DOI:** 10.7759/cureus.4564

**Published:** 2019-04-29

**Authors:** Ishrat Jahan, Salma Ahmed, Md Rabiul Islam, Abu Noim Md Abdul Hai, Mohammad Faijul Islam, Mohiuddin Ahmed Bhuiyan, Zabun Nahar

**Affiliations:** 1 Pharmacy, University of Asia Pacific, Dhaka, BGD; 2 Nephrology, National Institute of Kidney Diseases and Urology, Dhaka, BGD; 3 Nephrology, Holy Family Red Crescent Medical College Hospital, Dhaka, BGD

**Keywords:** orai1, genetic polymorphism, serum, calcium, phosphorus, ckd

## Abstract

Background

As chronic kidney disease (CKD) is a silent killer, it is now a global concern to find out the possible causes by genetic and biological markers. In the earlier stages of CKD, serum calcium and phosphorus levels fall down later on turned into hypercalcemia and hyperphosphatemia contributing high mortality in CKD. This study aimed to examine the serum calcium and phosphorus levels in non-dialysis CKD patients and healthy controls to find out their association with ORAI1 genetic polymorphism.

Methods

The present study recruited 96 non-dialysis CKD patients and 100 control subjects matched by age, gender, and body mass index (BMI). Measurement of serum calcium levels was performed with atomic absorption spectrophotometer (HITACHI, 170-30) and phosphorus levels were determined by UV VIS spectrophotometer (Analytik Jena SPEKOL 2000). PCR-RFLP technique was applied to determine the genetic polymorphism of ORAI1 (rs12313273 and rs6486795) gene.

Results

The mean values of serum calcium and phosphorus levels were 2.53 ± 0.50 mg/dL and 3.77 ± 0.42 mg/dL for the patient group and 3.67 ± 2.37 mg/dL and 13.66 ± 6.34 mg/dL for the control group, respectively. We observed significantly reduced serum calcium and phosphorus levels in non-dialysis CKD patients compared with control subjects (*p* < 0.001). No significant polymorphism of ORAI1 (rs12313273 and rs6486795) was found with declined serum calcium and phosphorus levels.

Conclusions

The present study suggested that there is no linear correlation between ORAI1 genetic polymorphism with serum calcium and phosphorus levels in non-dialysis CKD patients.

## Introduction

Chronic kidney disease (CKD) is a noteworthy reason for morbidity and mortality worldwide [1-2]. With the increasing prevalence around the globe, CKD has become a major public health problem. In America, approximately 11% to 15% of people have CKD [3]. According to Hill et al., the global prevalence of CKD is between 11% and 13%, and the majority of them are at stage 3 [4]. In Bangladesh, the data regarding CKD prevalence is scarce, whereas a recent study found that there was a high prevalence of CKD in urban middle-income Bangladeshis. They reported that one in every five middle-income urban Bangladeshis has CKD [5]. This number can be attributed to the fact that most of the traditional and non-traditional risk factors of CKD (diabetes, hypertension, and anemia) are seen in the majority of the people of Bangladesh.

The most prominent cause of death in CKD is cardiovascular complications [6-7]. Compared to the general population, cardiovascular disease is 10-20 folds higher in dialysis patients manifested by elevated serum calcium, hyperphosphatemia, secondary hyperparathyroidism, and ultimately vascular calcification contributing excess mortality in CKD [3,6,8-11]. For all the life-sustaining physiologic processes, tight control of calcium and phosphate homeostasis is mandatory [12]. Cell phosphate is considered as at the core of all energy-dependent physiologic process, whereas virtually all cellular functions such as modulation of the immune response, activation of inflammation, enzyme metabolism, muscle contraction, etc. are involved in the calcium-dependent pathway [12-13]. The capability of kidney controlling calcium varies according to different stages of CKD [14]. In dialysis patients, normal homeostasis of both calcium and phosphorus is compromised, resulting to hypercalcemia, hyperphosphatemia, elevated calcium phosphate products, high parathyroid hormone (PTH), and worsen secondary parathyroidism (SHPT) through a direct and indirect mechanism. However, in non-dialysis CKD patients, the association between serum phosphate levels and mortality has not been explored yet [10].

Genetic screening and linkage analysis in patients with defects in calcium release-activated calcium (CRAC) channel identified the association of ORAI1 [15]. ORAI1 is a tetra-spanning plasma membrane protein that forms the ion-conducting pore of CRAC channel. Recently, Hwang et al. demonstrated the association of ORAI1 gene polymorphism in the Taiwanese population by investigating five ORAI1 tSNPs (rs12313273, rs6486795, rs7135617, rs12320939, and rs712853). From their study, single nucleotide polymorphism of rs12313273 was found to be extensively linked with high serum calcium levels [13]. Besides, the association between inflammation and CKD has been explored in several studies. It is reported that racial variation is obvious for the susceptibility to renal disease and Indo-Asian origin have a greater risk of mounting end-stage renal disease than Caucasians [16]. There is no previous study concerning the association between genetic polymorphism of the ORAl1 gene and serum calcium and phosphorus levels among CKD patients in Bangladesh. The aim of this study was to examine the association of genetic polymorphisms of the ORAI1 gene with serum calcium and phosphorus levels in Bangladeshi non-dialysis CKD patients.

## Materials and methods

Study population

This case-control study enrolled in 96 non-dialysis CKD patients and one hundred healthy individuals. The patients were recruited from the National Institute of Kidney Diseases and Urology (NIKDU), Dhaka, Bangladesh and Department of Nephrology, Holy Family Red Crescent Medical College Hospital (HFRCMCH), Dhaka, Bangladesh. Qualified nephrologists diagnosed CKD patients by applying necessary procedures and laboratory tests. The healthy individuals were recruited from the same catchment area matched by age, gender, and body mass index (BMI). These study participants were free from any other severe illness. All subjects were assessed through a detail medical history to exclude any systemic diseases that can interfere with the serum calcium or phosphorus levels or genetic polymorphisms of the ORAI1 gene. Additional exclusion criteria were alcohol or substance abuse, mental retardation, psychiatric illness, and the presence of infectious disease.

Blood sample collection

Blood samples (5 ml) were drawn from the cephalic vein of each subject using a syringe fitted with a stainless steel needle. For serum calcium and phosphorus analysis, the collected blood was allowed to clot for at least one hour at room temperature and then centrifuge at 1000 x g 15 minutes to separate the serum. The serum samples were placed into microtubes and stored at -80 °C until quantitative analysis. On the other hand, for genetic analysis, the blood samples were transferred within vacuum tubes containing EDTA (ethylenediaminetetraacetic acid) and stored into the refrigerator at -20 °C.

DNA extraction and quantification

Genomic DNA was extracted by blood genomic DNA extraction mini kit (Favorgen, Taiwan) as per the manufacturer’s instruction. The concentration of extracted DNA was measured using a microvolume spectrophotometer (Genova Nano, Jenway). The extracted DNA sample (2 µl) was transferred to the Genova Nano micro-volume spectrophotometer and concentration of DNA in microgram per milli-liter was measured. To assess the purity of DNA, the ratio of absorbances was taken at 260 nm and 280 nm.

Quantification of serum calcium and phosphorus

Measurement of serum calcium levels was performed with atomic absorption spectrophotometer (HITACHI, 170-30) and phosphorus levels were determined by UV VIS spectrophotometer (Analytik Jena SPEKOL 2000). Briefly, collected samples were diluted with deionized water and the dilution factor was 10. Calibration curve was prepared from the different concentrations (0.5, 1.0, 5.0, 10.0 and 12.0 mg/L) of standard calcium and phosphorus. Finally, the concentrations of calcium and phosphorus were measured by reading the absorbance at 422.7 nm for calcium and 710 nm for phosphorus, respectively. The test quality and precision were confirmed by running one standard solution for every 10 test samples. Five blank solutions were analysed to establish the limits of detection (LoDs) for serum calcium and phosphorus. Microsoft office Excel 2010 program was run to determine the σ value. LoDs were observed as 1.5 μg/L for calcium and 0.01 μg/L for phosphorus. Serum calcium and phosphorus levels were calculated by SpectrAA software package using the calibration curve.

Genotyping

Two tagging SNPs of ORAI1 (rs12313273 and rs6486795) with a minor allele frequency (MCFs) of >10%were selected from HapMap database. The PCR-RFLP method was applied to determine the ORAI1 gene polymorphisms. The fragments of ORAI1 were amplified by PCR using forward primers of 5’-AACGCTCCAACAGCCAAATA-3’ and reverse primers of 5’-GGAGAGGGGCAGGAAATAAG-3’ and another forward primer of 5’-ACGCCTGGACTGAGAAGTAC-3’ and reverse primers of 5’-TAAAGAACGCCAGGCCAAAC-3’, followed by gel electrophoresis for visualization. DNA ladders (50bp and 100bp) were also used for size estimation of all restriction enzyme digestion fragments, allowing for precise and reliable genotyping of samples.

Statistical analysis

Independent sample *t*-test and cross table variables were used to compare all the parameter of non-dialysis CKD patients and controls. Mann-Whitney U test and Fisher’s exact test were performed to evaluate differences between the groups. The linear association between variables was assessed by the Spearman's rank correlation coefficient test. For all the tests p ≤ 0.05 was considered statistically significant. Statistical Package for Social Sciences (SPSS) version 23.0 (IBM Corp., Armonk, NY) was used to perform all those tests.

## Results

The distribution of characteristics and laboratory findings of participants are presented in Table 1.

**Table 1:** Characteristics and clinical outcomes of the study population Significant *p*-values ≤ 0.05 at 95% confidence interval; values in bold: *p* < 0.05 BMI, body mass index; SD, standard deviation; N, number

Parameters	Patients (N = 96)	Controls (N = 100)	p-value
Age in years, mean ± SD	45.46 ± 12.29	42.76 ± 12.60	0.202
Gender, male/female	50/46	53/47	0.631
BMI (kg/m^2^), mean ± SD	26.51 ± 2.02	26.26 ± 2.62	0.518
Smoker (%)	34%	30%	0.362
Serum calcium (mg/dL)	2.53 ± 0.50	3.77 ± 0.42	<0.001
Serum phosphorus (mg/dL)	3.67 ± 2.37	13.66 ± 6.34	<0.001

The controls and the patients were recruited as similar age, gender and BMI with no significant variation (*p* ≥ 0.05), whereas in the laboratory analysis, there were significant differences (*p* ≤ 0.05) of serum calcium and phosphorus between patients and control group. Significantly reduced levels of calcium and phosphorus were observed in non-dialysis CKD patients compared to healthy individuals. A significant positive correlation (*r* = 0.582; *p* < 0.001) was observed between serum calcium and phosphorus levels in non-dialysis CKD patients. Two tagging SNPs of ORAI1 (rs12313273 and rs6486795) with a minor allele frequency were selected from the HapMap database. In both cases, genetic analysis revealed a maximum number of TT genotype, normal homozygous with a few heterozygotes. However, no significance mutation was shown in respective SNPs (Table 2). 

**Table 2:** Genotyping and allele frequency of ORAI1 gene among study population Significant *p*-values ≤ 0.05 at 95% confidence interval N, number

	Genotype	Allele	Patients (%) (N = 96)	Controls (%) (N = 100)	Genotype p-value	Allelic p-value
	TT	T	89 (92.7)	99 (99.0)		
rs12313273	CT	C	5 (5.2)	1 (1.0)	0.462	0.532
	CC		2 (2.1)	0 (0.0)		
	TT	T	91 (94.8)	100 (100.0)		
rs6486795	CT	C	4 (4.2)	0 (0.0)	0.173	0.361
	CC		1 (1.0)	0 (0.0)		

Associations of the ORAI1 gene with altered serum calcium and phosphorus levels in CKD patients are presented in Table 3. 

**Table 3:** Difference in the calcium and phosphorus levels in non-dialysis CKD patients stratified by different ORAI1 genotype Significant *p*-values ≤ 0.05 at 95% confidence interval CKD, chronic kidney disease; N, Number

	Genotype	Patients (%) (N = 96)	Calcium (mg/dL)	p-value	Phosphorus (mg/dL)	p-value
	TT	89 (92.7)	2.59 ± 0.52		3.67 ± 0.83	
rs12313273	CT	5 (5.2)	2.40 ± 0.17	0.354	2.38 ± 0.36	0.211
	CC	2 (2.1)	2.48 ± 0.81		3.41 ± 0.91	
	TT	91 (94.8)	2.59 ± 0.62		3.68 ± 0.42	
rs6486795	CT	4 (4.2)	2.37 ± 0.16	0.243	2.48 ± 0.46	0.152
	CC	1 (1.0)	2.62 ± 0.00		3.23 ± 0.00	

## Discussion

Latest studies on the genetic susceptibility and the development of CKD have yielded promising results. The results of a genome-wide association study showed that several loci were associated with CKD and estimated glomerular filtration rate (eGFR) [17]. A major correlation was found between TRPC1, ORAI1, STIM1 and parathyroid cells [13,18]. Some recent studies in Asia revealed the significance of the ORAI1 gene in calcium regulation among CKD patients. Chou et al. demonstrated an association of ORAI1 with the risk and recurrence of calcium nephrolithiasis in Taiwanese people [19]. Another study in the same region found that ORAI1 polymorphism was linked with increased serum calcium [13]. Mineral abnormalities such as elevated calcium, phosphorus, and PTH are found in non-dialysis CKD patients that later on contributing increased death in CKD patients [20-22]. However, there was no previous study investigating the influence of calcium and phosphorus regulating channels in case of CKD patients in Bangladesh. As we also belong to an Asian country, we conducted this study in order to observe if there was any similarity pattern between our outcomes and previous findings or not.

In our study, after genotyping of SNPs of ORAI1 (rs12313273 and rs6486795), no mutation was found rather than some minor heterozygotes. On the other hand, the results of serum calcium and phosphorus were found in much-declined levels compared to healthy subjects. It is already established that low levels of calcium, phosphorus in early staged CKD patients eventually turned into hypercalcemia and hyperphosphatemia which is directly linked to vascular calcification- the most frequent cause of death in CKD [3,6,10].

Calcium influxes in T cells through the CRAC channels whereas ORAI1 is essential for the function of CRAC channels [23-27]. From the recent study of Hwang et al, five ORAI1 tagging SNPs such as rs12313273, rs6486795, rs7135617, rs12320939, and rs712853 were investigated among Taiwanese CKD patients [13]. None of the tSNPs of ORAI1 were associated with the risk of CKD except polymorphism of rs12313273 ORAI1 gene which was significantly associated with elevated serum calcium levels. As our study revealed a significant decrease of serum calcium and phosphorus level in non-dialysis CKD patients, we expected some linear correlation with ORAI1 thus found no significant mutation of rs12313273 and rs6486795.

There were some limitations in our study. First, we did not consider other factors that can be correlated with serum calcium levels and other comorbidities were not identified during sample collection. Second, the sample size was little and we recruited only the urban population which cannot be the representative of the whole population. Our results revealed that the association of ORAI1 genotype with calcium and phosphorus levels as we found neither elevation of calcium level nor the alteration of these SNPs. That is why extensive studies with a large population are needed to fully understand the linkage between ORAI1 and altered calcium and phosphorus levels.

## Conclusions

To the best of our knowledge, this is the first study in Bangladeshi CKD patients examining ORAI1 gene polymorphism with serum calcium and phosphorus. Though our study yields no correlation, further replication studies with large cohort should be carried out to clarify the effect of ORAI1 genetic polymorphism in CKD.
